# War on Ukraine: Impact on Ukrainian Medical Students

**DOI:** 10.5195/ijms.2022.1468

**Published:** 2022-04-05

**Authors:** Bahadar Singh Srichawla, Mohammad Amin Khazeei Tabari, Mihnea-Alexandru Găman, Alejandro Munoz-Valencia, Francisco J. Bonilla-Escobar

**Affiliations:** 1 DO MS. Resident Physician, UMass Chan Medical School Department of Neurology, United States. Student Editor, IJMS.; 2 Medical student, Student Research Committee, Mazandaran University of Medical Sciences. USERN Office, Mazandaran University of Medical Sciences, Sari, Iran. Associate Editor, IJMS.; 3 MD, PhD student. Faculty of Medicine, “Carol Davila” University of Medicine and Pharmacy, 050474 Bucharest, Romania & Department of Hematology, Center of Hematology and Bone Marrow Transplantation, Fundeni Clinical Institute, 022328 Bucharest, Romania. Scientific Editor, IJMS.; 4 MD, PhD student. Institute for Clinical Research Education (ICRE), Department of Surgery, Global Surgery, University of Pittsburgh, Pittsburgh, PA, United States.; 5 MD, MSc, PhD(c). Researcher, Department of Ophthalmology; Institute for Clinical Research Education (ICRE), University of Pittsburgh, Pittsburgh, PA, United States. CEO, Fundación Somos Ciencia al Servicio de la Comunidad, Fundación SCISCO/Science to Serve the Community Foundation, SCISCO Foundation, Cali, Colombia. Grupo de investigación en Visión y Salud Ocular, VISOC, Universidad del Valle, Cali, Colombia. Editor in Chief, IJMS.

The ongoing Russian invasion of Ukraine has taken a tremendous toll on the physical and mental wellbeing of the Ukrainian people. Accordingly, medical trainees and institutions must adapt to a high degree of uncertainty and turmoil. The first medical school in Ukraine was the Collegium Medicum founded in 1773 in Lviv. With the fall of the Soviet Union in 1991, Ukraine had 14 medical institutes within its borders aimed at teaching students medical and pharmaceutical sciences^1^. In 2022, Ukraine has 23 medical institutions filled with not only Ukrainian nationals but medical students from around the world. It is estimated that approximately 18,000 students from India alone study in Ukraine, many of whom are medical learners. Medical degrees earned at Ukrainian institutions are recognized throughout the world, including by the World Health Organization (WHO) and European Council. Ukraine has accepted many foreign medical students who could not gain entry in their home countries for political reasons as well as those who could not afford the high price to study medicine in their home country.^2^ Additionally, Ukrainian medical institutions already hosting a diverse array of trainees have had to adjust to the ongoing COVID-19 pandemic which has had a negative impact on medical education worldwide. Medical schools have resorted to virtual education and many students have been pulled out of clinical rotations for extended periods.^3^ This stress on medical students has not been exponentially compounded by the reality of war.

Many of these national and foreign medical students are now displaced refugees looking to escape to Western Europe or their country of origin. Even by escaping the ongoing conflict, the question remains as to how many of these students will complete their medical education and cope with the trauma of political unrest. Officials from medical universities in India have indicated accommodations will be made, however no concrete plan is currently in place. The careers of thousands of medical students remain in the balance. Although many students have been able to obtain refugee status many still await admission on the border of neighboring countries. Not only do they have to worry about the education and career, but the safety of their peers and families. Because of the ongoing conflict many of these foreign students will likely pursue their medical degrees in other countries such as Italy, Spain, and Germany. Unfortunately, emerging reports indicate that some of these emerging leaders in medicine have had their lives prematurely ended in the ongoing shelling of Ukrainian cities.^4^ It is a true tragedy and loss to the medical community that these young students who have dedicated themselves to a field of helping others are will now never be able to realize their potential. Many of these bombardments have destroyed the key infrastructure of medical universities throughout Ukraine. The long-term effects on medical education in Ukraine are catastrophic with the lack of resources and infrastructure to support it. Tsagkaris et al. classified the consequences of the aforementioned war into four categories: physical injury and mental health consequences to Ukrainians (not only soldiers but also civilians); destruction of healthcare establishments; destruction of non-healthcare critical infrastructure; and impact on the environment (via the use of toxins and/or nuclear radiation during the war).^5^ Medical students and healthcare workers will suffer tremendously from the consequences of war.

It is likely many medical students will be diverted from their studies to work on the front lines, and their medical education will be halted in Ukraine indefinitely. As the crisis continues, medical education that does occur may be restricted to online classes and will have a negative academic impact on students. Creating an environment where medical students have a stable internet connection and the resources to access their courses is essential and difficult to prioritize in these circumstances. An example of this type of scenario occurred in Iraq, 18 years ago. The political unrest in Iraq in 2003 had a negative impact on medical education. Frequent threats and attacks, accompanied by declining social order, led to the emigration of most medical professors from Iraq. This forced migration of medical professors had a great adverse effect on the leadership of the medical education system.^6^

The International Federation of Medical Students’ Associations (IFMSA) and medical students from across the globe are calling for an immediate halt to the violence and the restoration of peace in Ukraine.^7^ The possibility of enormous fatalities, physical damage, and relocation of citizens concerns the Ukrainians greatly. As the Russian invasion of Ukraine moves into the fifth week the stress on the Ukrainian medical system is unprecedented. From waning medical supplies, to the lack of critical personnel including doctors and nurses, and direct assault on hospital infrastructure itself, critical intervention is needed. Willing medical students may choose to serve as frontline workers in Ukrainian hospitals. Displaced Ukrainian medical students may also serve in a safer environment, setting up relief efforts in the neighboring nations of Romania, Poland, and Hungary where many refugees have fled to. We ask medical students, health care workers, and young people around the world to stand up for peace and follow the principles of humanity, neutrality, and impartiality in their work and communication.

This is the first time that the International Journal of Medical Students Editorial Team has commented on a political conflict. We have learned in our tenure that medical students and their education worldwide is affected by many factors, including global warming and conflict. We have raised our voice before in favor of actions to prevent climate change.^9–11^ We are adding our voice of support to those suffering from political unrest and acts of violence globally, with specific focus on the Ukraine.^12^ The path of war and vengeance is an easy one when compared to political dialogue and collaboration. We, the new generation of scientists of the world, claim for the use of reason over emotions to keep us all safe and promote progress worldwide.
